# Supplementary material for: Integrative Proteomics and Phosphoproteomics Analysis of the Rat Adenohypophysis after GnRH Treatment
Source: Int J Mol Sci. 2023 Feb 7;24(4):3339. doi: 10.3390/ijms24043339 (PMC9961725; doi:10.3390/ijms24043339)
Supplement: Supplementary file 1 [file ijms-24-03339-s001.zip › Supplementary Figures.pdf]

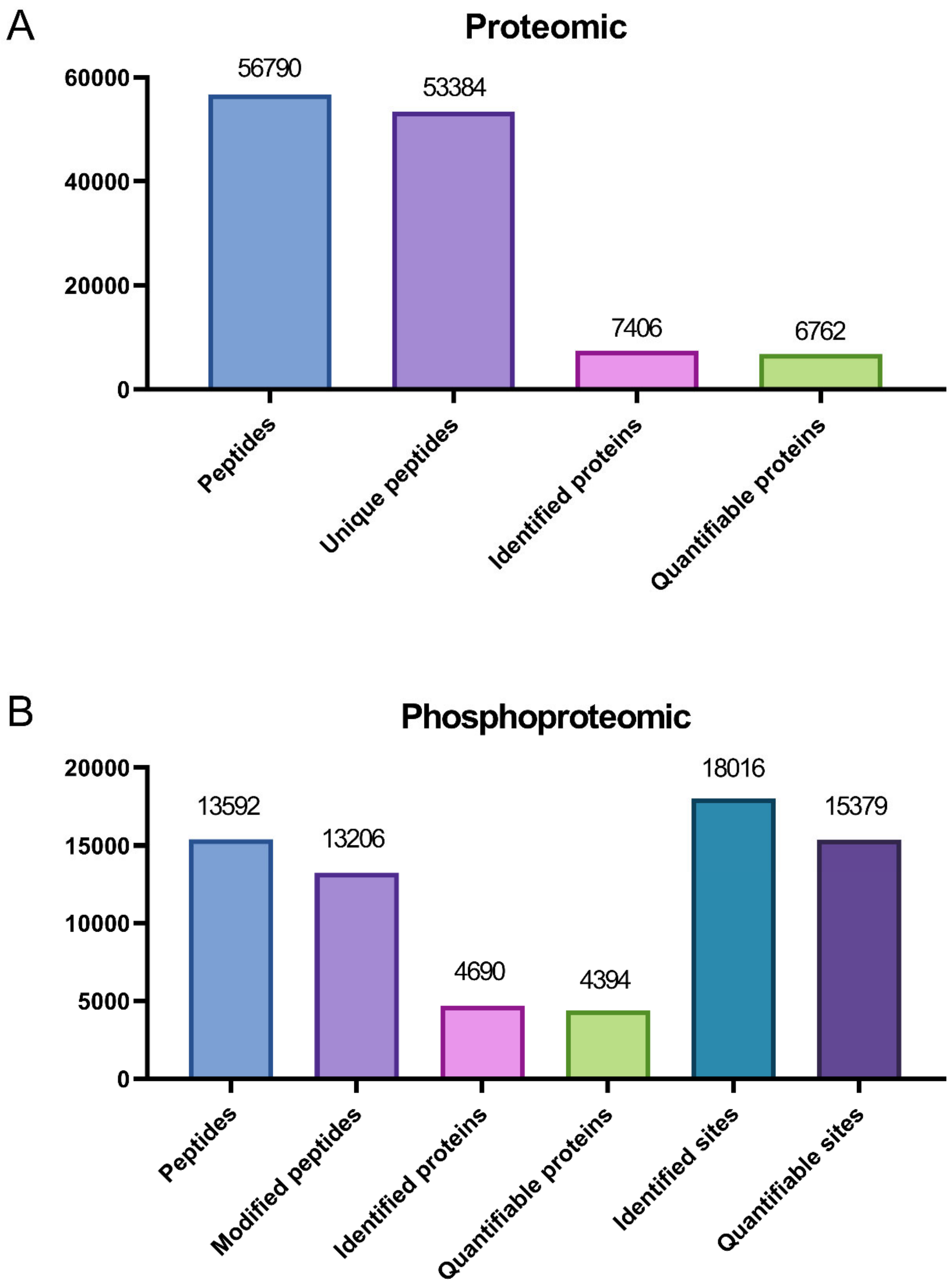

Figure S1. Basic statistics of mass spectrometry data in proteomics(A) and phosphoproteomics(B).

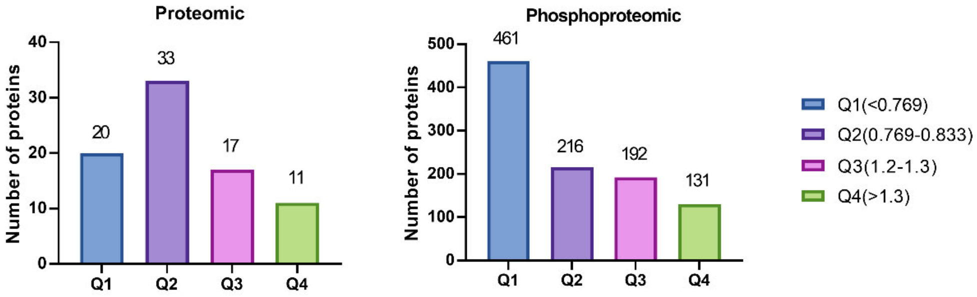

Figure S2. Distribution of the number of differentially expressed proteins and differentially modified sites in the Q1-Q4 groups.

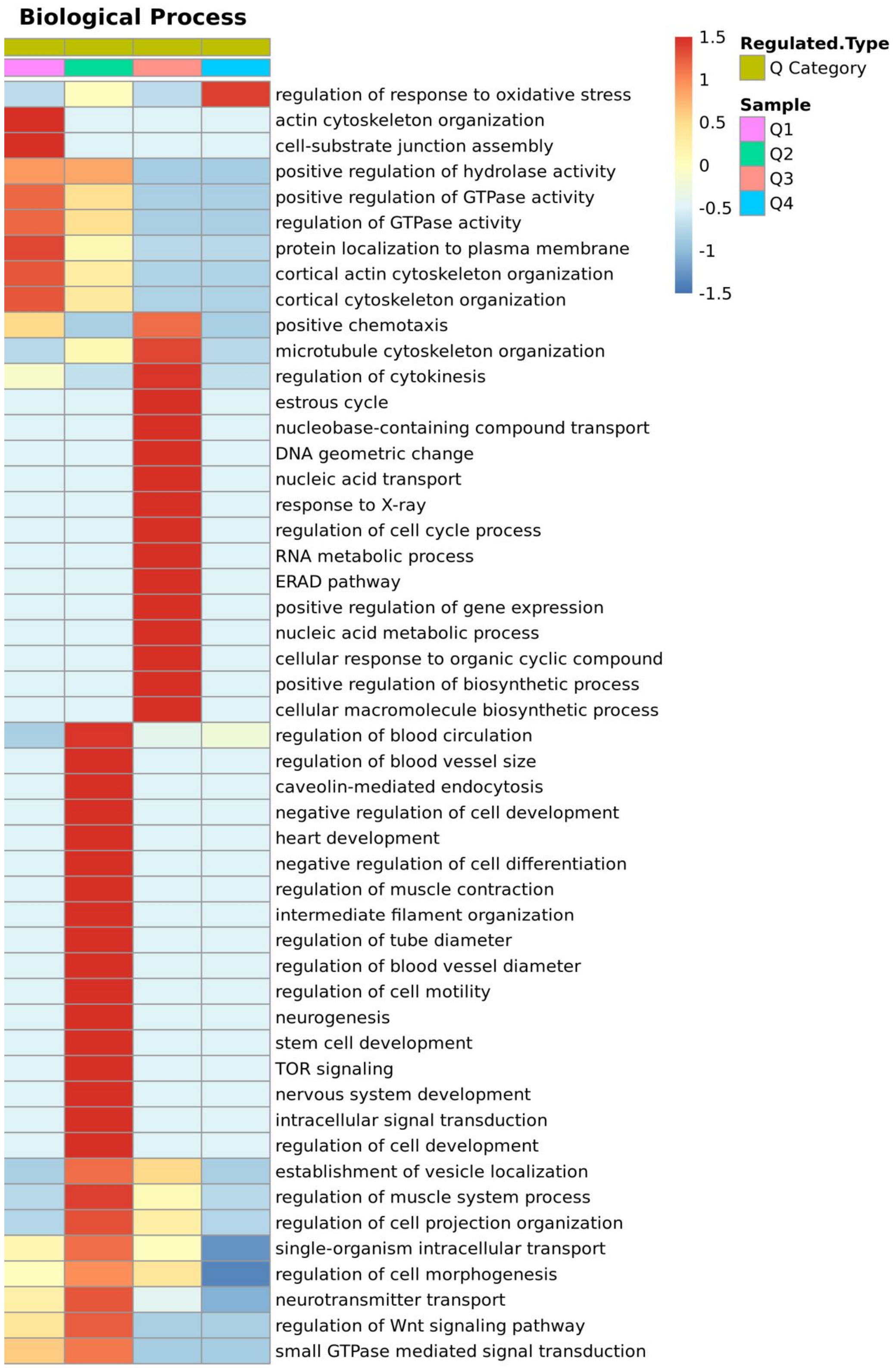

Figure S3. Heatmap for cluster analysis of DEPPs based on the biological process module of GO classification.

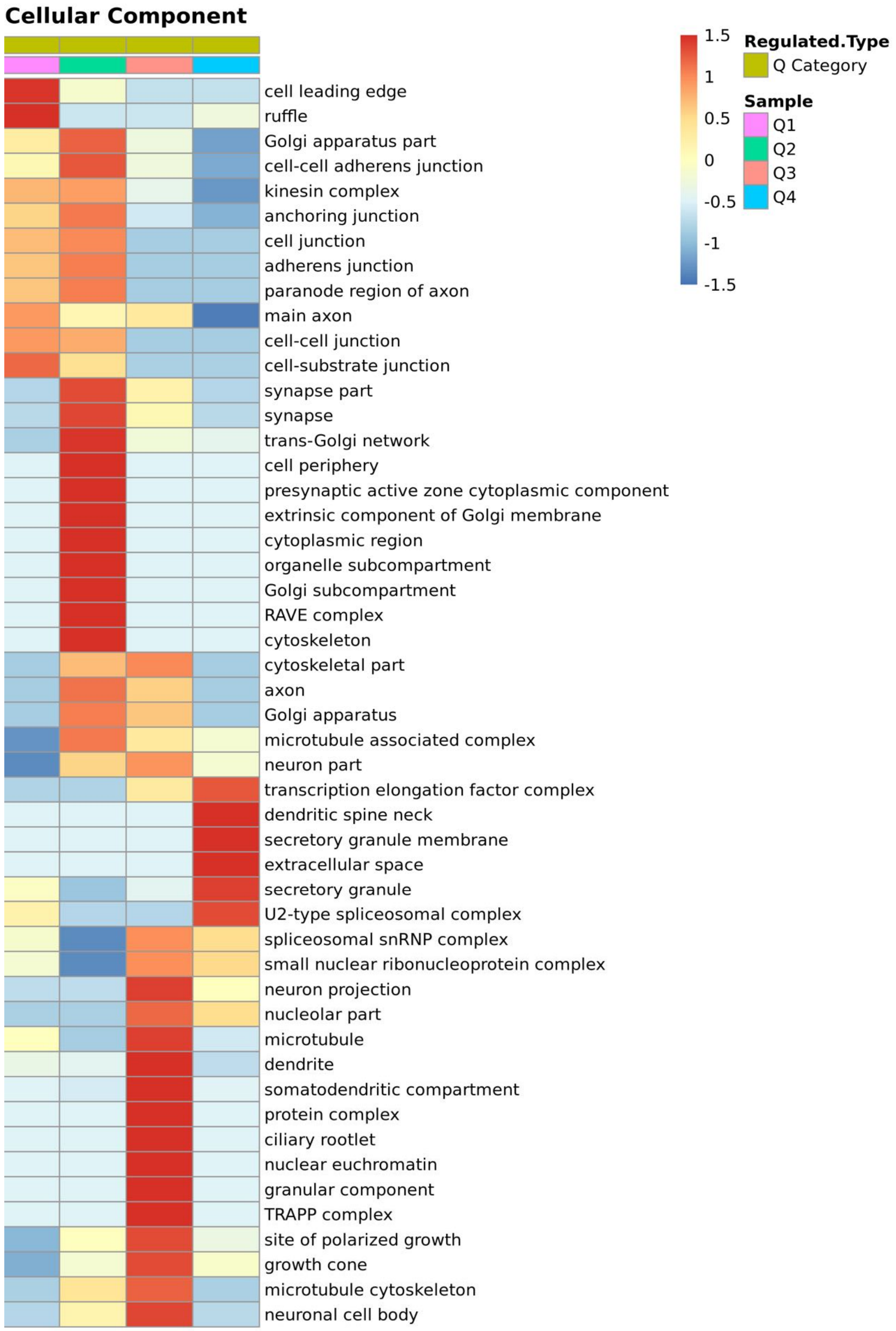

Figure S4. Heatmap for cluster analysis of DEPPs based on the cellular component module of GO classification.

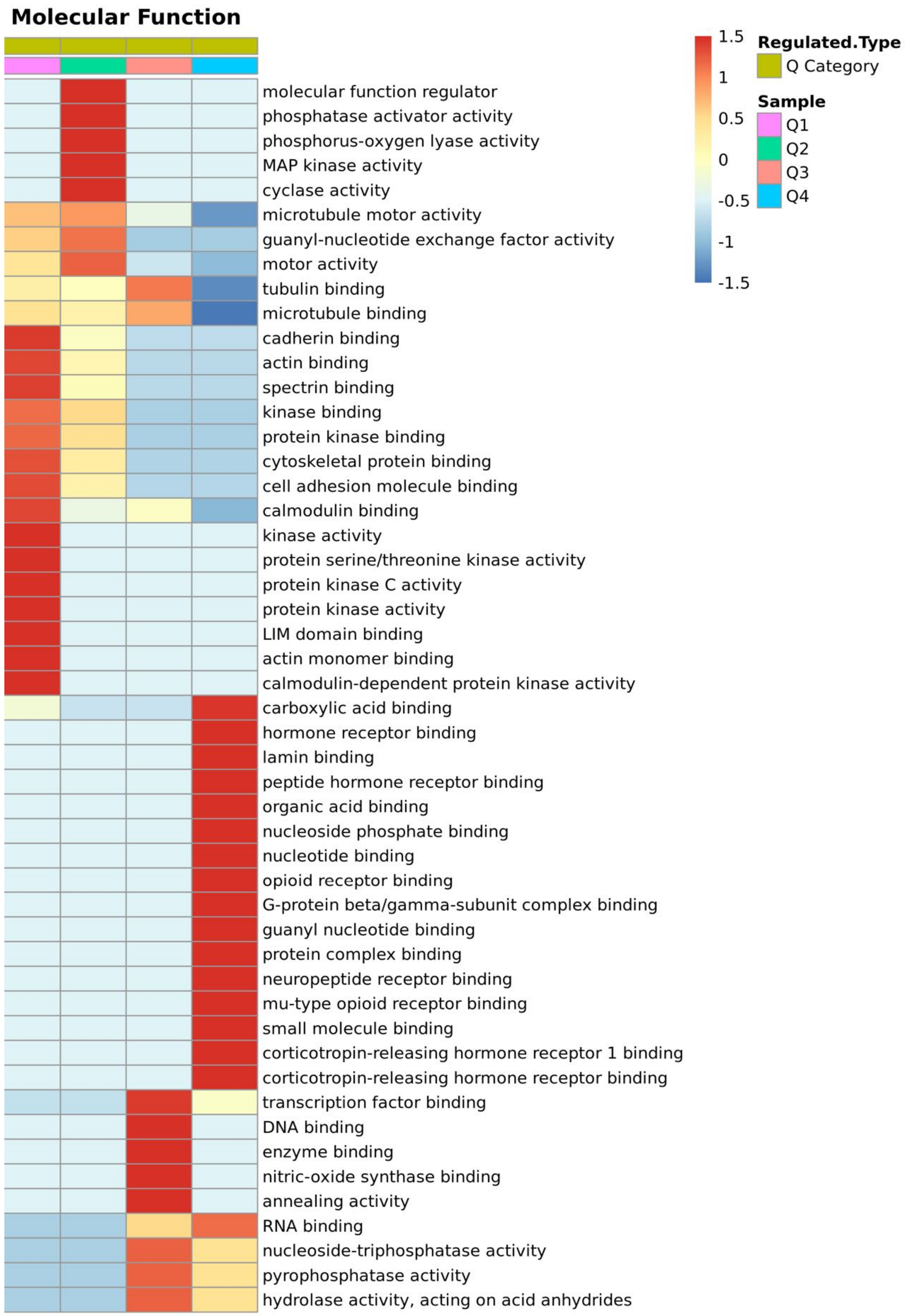

Figure S5. Heatmap for cluster analysis of DEPPs based on the molecular function module of GO classification.

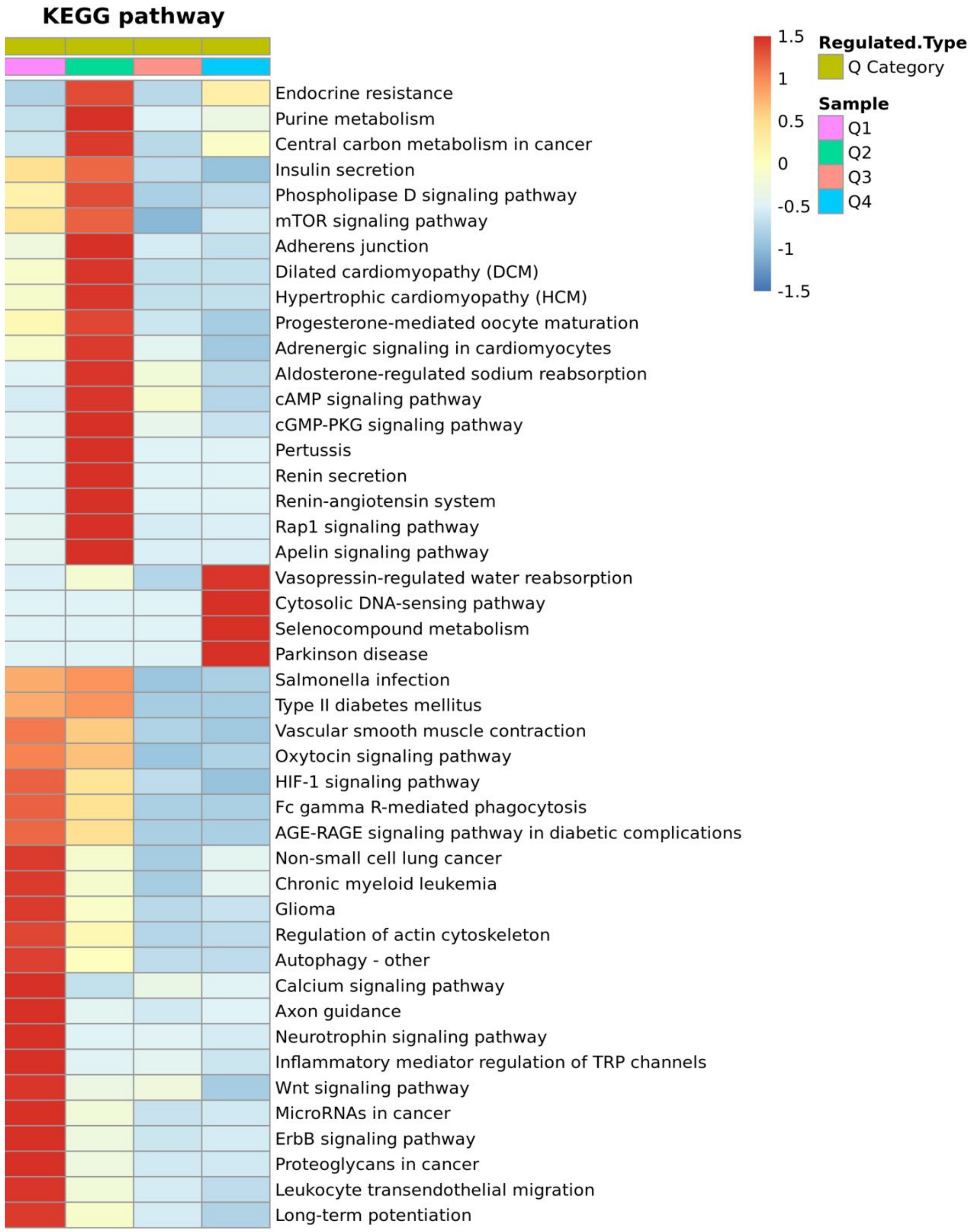

Figure S6. Heatmap for cluster analysis of DEPPs based on KEGG pathway analysis.

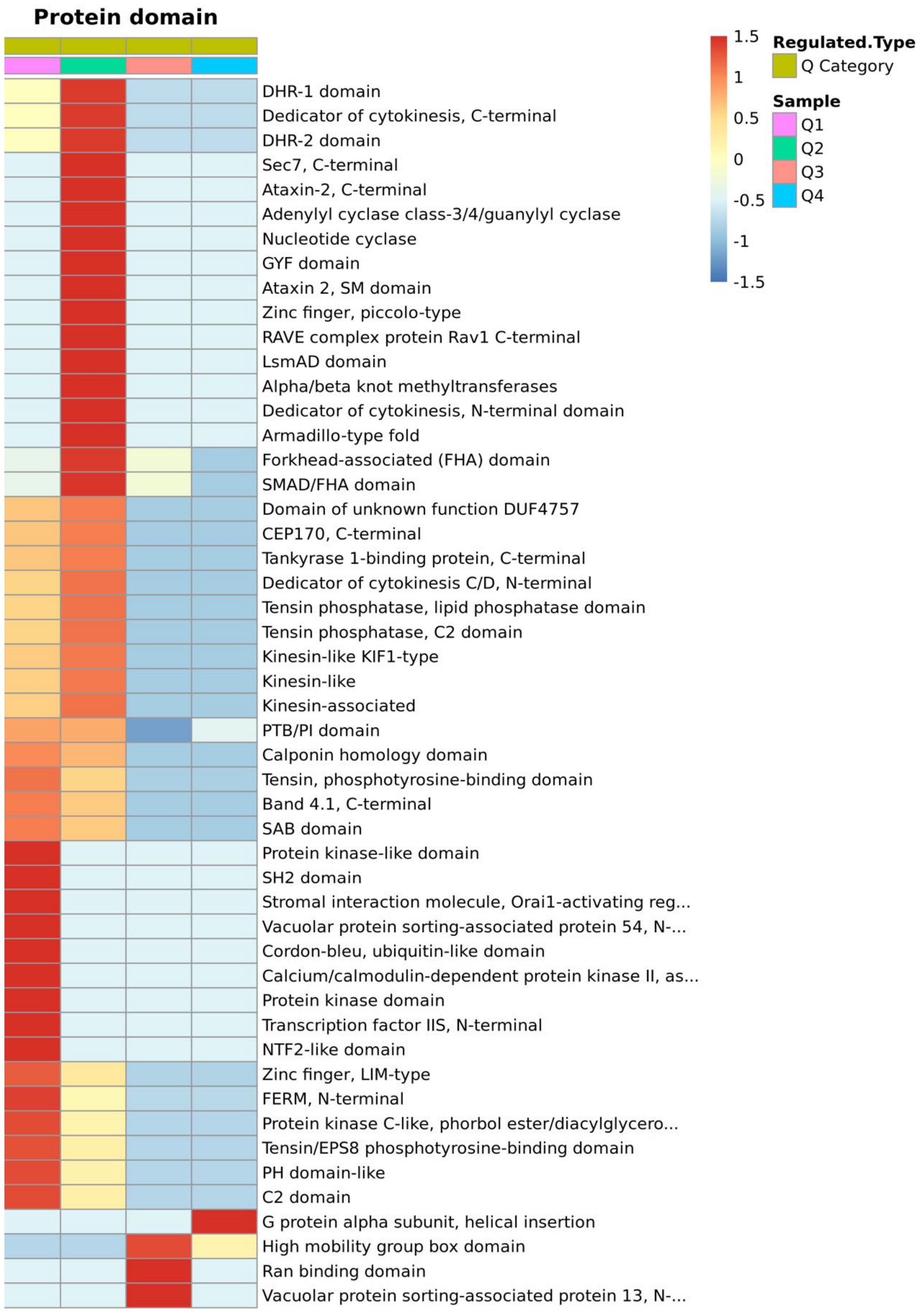

Figure S7. Heatmap for cluster analysis of DEPPs based on protein structural domain enrichment analysis.
